# Strengthening Mechanism of Titanium Boride Whisker-Reinforced Ti-6Al-4V Alloy Matrix Composites with the TiB Orientation Perpendicular to the Loading Direction

**DOI:** 10.3390/ma12152401

**Published:** 2019-07-28

**Authors:** Hiroki Kurita, Shiori Suzuki, Shoichi Kikuchi, Noriharu Yodoshi, Sophie Gourdet, Fumio Narita

**Affiliations:** 1 Department of Materials Processing, Graduate School of Engineering, Tohoku University, Sendai 980-8579, Japan; 2 Department of Mechanical Engineering, Faculty of Engineering, Shizuoka University, Hamamatsu 432-8561, Japan; 3 Cooperative Research and Development Center for Advanced Materials (CRDAM), Institute for Materials Research (IMR), Tohoku University, Sendai 980-8577, Japan; 4 Ariane Group, 78130 Les Mureaux, France

**Keywords:** titanium matrix composite, tensile property, whisker, strengthening mechanism

## Abstract

We fabricated fully dense titanium boride (TiB) whisker-reinforced Ti-6Al-4V alloy matrix (Ti6Al4V-TiB) composites, with a homogeneous dispersion, a TiB orientation perpendicular to the loading direction (; two-dimensional random direction) and an intimate Ti/TiB interface without an intermediate interfacial layer in the Ti-6Al-4V alloy matrix, by spark plasma sintering. Microstructural analysis allows us to present the tensile properties of the Ti6Al4V-TiB composites with the theories for discontinuous fiber-reinforced composites. The Ti6Al4V-TiB 10 vol.% composite yielded a Young’s modulus of 130 GPa, an ultimate tensile strength (UTS) of 1193 MPa and an elongation of 2.8%. The obtained experimental Young’s modulus and UTS of the Ti6Al4V-TiB composites were consistent with the theoretical values estimated by the Halpin-Tsai and Shear-lag models. The good agreement between our experimental results and these models indicates that the TiB whiskers behave as discontinuous fibers in the Ti-6Al-4V alloy matrix.

## 1. Introduction

Titanium (Ti) alloys are widely used as aerospace materials due to their high specific strengths and corrosion resistance. Moreover, the remarkable compatibility of Ti alloys with carbon fiber-reinforced plastics, which have recently come into use as structural materials for aircrafts, has led to an increase in the demand for the development of new Ti materials that possess higher specific strengths than conventional Ti alloys to further improve fuel efficiency. Among the available Ti materials, Ti matrix composites (TMCs) have attracted the most attention due to their potential for possessing higher specific mechanical properties than conventional Ti alloys [1,2]. Silicon carbide (SiC), aluminum oxide (Al_2_O_3_), silicon nitride (Si_3_N_4_) and boron carbide (B_4_C) have been considered as reinforcements for TMCs; however, the chemical compounds between these reinforcements and the Ti alloy at the interface often destabilize the TMCs [1,2,3,4]. It has been reported that titanium boride (TiB) whiskers can be formed in Ti alloys by the following chemical reaction:

(1) $${\mathrm{Ti}+\mathrm{TiB}}_{2}\to 2\mathrm{TiB}$$

and that these TiB whiskers are chemically stable in the Ti alloys [1,2]. Furthermore, it seems that the slight difference in the thermal expansion coefficient between TiB (7.15 × 10^−6^ K^−1^) and Ti (8.6 × 10^−6^ K^−1^) [2,3] allows some of the remaining thermal stress inside the TiB whiskers to be dispersed within the Ti alloy matrix (Ti-TiB) composites. Therefore, it is likely that TiB can be a prime candidate for the reinforcement of TMCs [5,6,7].

Previous studies have reported that the existence of TiB whiskers causes the embrittlement of Ti alloys, while Ti-TiB composites have outstanding tensile strengths [8,9,10,11,12,13]. However, the agglomeration and incomplete precipitation of TiB whiskers in the Ti alloy matrix still inhibit our understanding of the relationship between the tensile properties and microstructure of Ti-TiB composites. Consequently, it has not been achieved neither understanding the strengthening mechanism of Ti-TiB composites yet, nor designing the microstructure of Ti-TiB composites to discuss their strengthening mechanism by the theories, to the best of our knowledge.

In this study, we fabricated TiB whisker-reinforced Ti-6Al-4V alloy matrix (Ti6Al4V−TiB) composites, which have an applicable microstructure for the equations for discontinuous fiber-reinforced composites. We evaluated the tensile properties of these Ti6Al4V-TiB composites and compared them with the theoretical values estimated by the equations for discontinuous fiber-reinforced composites to reveal their strengthening mechanism.

## 2. Experimental Procedure

We prepared a spherical gas-atomized Ti-6Al-4V powder (TILOP64-45, OSAKA Titanium Technology Co., Ltd., Amagasaki, Japan), with a particle size of less than 45 μm, and a spherical titanium diboride (TiB_2_) powder (TiB_2_-NF, Japan New Metals Co., Ltd., Toyonaka, Japan), with a particle size of 1–2 μm, as the starting materials. After mixing the powder in air for 10 min by hand, we put the mixed powder into a graphite die, and consolidated at 800, 900, 1000 and 1100 °C for either 30 or 60 min under a uniaxial pressure of 10 MPa in a spark plasma sintering (SPS) machine (Dr. Sinter SPS-1050, Fuji Electric Industrial Co., Ltd., Tsurugashima, Japan). The quantity of TiB_2_ powder was controlled to obtain TiB volume fractions of 7.5, 10 and 12.5 vol.% in the final Ti6Al4V-TiB composites. The vacuum level was maintained at 5–6 Pa during SPS. The powders were heated at a rate of 50 °C/min. We inserted a graphite sheet, with a thickness of 0.2 mm, between the carbon die and mixed powder to avoid adhesion between the carbon die and the obtained Ti6Al4V-TiB composites after SPS. The temperature was monitored on the surface of the carbon die with a radiation thermometer, which was positioned in front of the glass porthole of the SPS machine. The Ti6Al4V-TiB composites were then cooled by furnace cooling. The size of the obtained Ti6Al4V-TiB composites was *ϕ* 50 × 2 mm^2^ for microstructural observation. For tensile tests, we fabricated Ti6Al4V-TiB composites, with a thickness of less than 1 mm, at 1100 °C for 60 min, to discuss their strengthening mechanism with the TiB orientation perpendicular to the loading direction (two-dimensional random direction) in the Ti matrix. We also consolidated a Ti-6Al-4V alloy (i.e., a Ti6Al4V-0 vol.% TiB composite) at 1100 °C for 60 min for comparison with the tensile properties and microstructure of the Ti6Al4V-TiB composites. 

The chemical compositions of the Ti6Al4V-TiB composites were identified with X-ray diffraction (XRD; RINT Ultima II, Rigaku Corporation, The Woodlands, TX, USA) and a field emission electron probe micro analyzer (FE-EPMA; JXA-8530F, JEOL Ltd., Tokyo, Japan). The XRD patterns were obtained with a counting time of 4/s, a step size of 0.02° using CuKα radiation, a voltage of 40 V and a current of 40 A. We determined the scanning range between 30° and 50°, with Ti, TiB_2_, and TiB having peaks at different angles, by the powder data files from the International Centre for Diffraction Data for 00-044-1294 (Ti), 00-035-0741 (TiB_2_) and 00-006-0641 (TiB) [14,15,16]. The acceleration voltage of the FE-EPMA was 15 kV. The microstructure of the Ti6Al4V-TiB composites was imaged with a field emission scanning electron microscope (FE-SEM; JSM-7001F, JEOL, Tokyo, Japan), operating at an acceleration voltage of 15 kV and using a back-scattered electron detector. 

**Figure 1:**
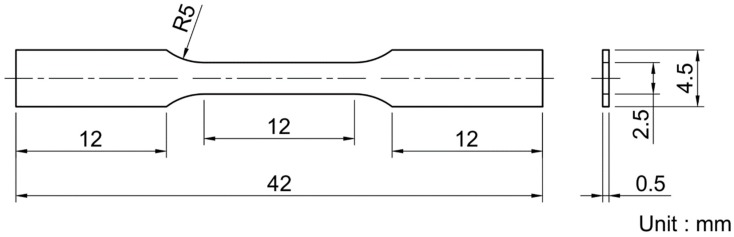
Dimensions of the specimens used in the tensile tests.

We prepared the tensile test specimens with the dimensions shown in Figure 1 from the fabricated Ti6Al4V-TiB composites by wire-cut electrical discharge machining (EDM, MV1200R, Mitsubishi Electric Corporation, Tokyo, Japan). The surface of the specimens was polished by waterproof abrasive papers with #600 roughness to remove the surface layer that was altered by the wire-cut EDM. Following ISO 6892-1 (“Metallic materials-Tensile testing-Part 1: Method of test at room temperature”), the tensile properties of the specimens were investigated using a material testing machine (Ag-50kN Xplus, Shimadzu Corporation, Kyoto, Japan) at room temperature and a cross-head speed of 0.5 mm/min. The strain of the specimens was monitored using a strain gauge (KFGS-1-120-C1-11, Kyowa Electronic Instruments Co., Ltd., Higashine, Japan) with a 1-mm gauge length and a nominal resistance of 120 Ω, which was positioned at the center of the specimens. The elongated part of the specimens was marked with two dots to evaluate the elongation of the specimens after the tensile tests. The fracture surface of the specimens was imaged with an FE-SEM (JSM-7001F, JEOL), operating at an acceleration voltage of 15 kV and using a back-scattered electron detector.

## 3. Models 

### 3.1. Halpin-Tsai Model

Halpin and Tsai proposed an equation to estimate the Young’s modulus for two-dimensional randomly oriented discontinuous fiber-reinforced composites, which is defined as [1,17]:

(2) $$E_{c}=\frac{3}{8}E_{11}+\frac{5}{8}E_{22}$$

where:

(3) $$E_{11}=\frac{1+2\left(\frac{l_{f}}{d_{f}}\right)\eta_{L}V_{f}}{1-\eta_{L}V_{f}}E_{m}$$

(4) $$E_{22}=\frac{1+2\eta_{T}V_{f}}{1-\eta_{T}V_{f}}E_{m}$$

(5) $$\eta_{L}=\frac{\left(\frac{E_{f}}{E_{m}}\right)-1}{\left(\frac{E_{f}}{E_{m}}\right)+2\left(\frac{l_{f}}{d_{f}}\right)}$$

and

(6) $$\eta_{T}=\frac{\left(\frac{E_{f}}{E_{m}}\right)-1}{\left(\frac{E_{f}}{E_{m}}\right)+2}$$

E and V are the Young’s modulus and volume fraction, respectively. The longitudinal and transverse moduli are indicated by $E_{11}$ and $E_{22}$, respectively. The subscripts c, f and m indicate the composite, fiber and matrix, respectively. l and d are the length and diameter of the fiber, respectively, where $l_{f}/d_{f}$ is the aspect ratio of the fiber.

### 3.2. Shear-Lag Model

Kelly and Tyson proposed that the ultimate tensile strength (UTS) of oriented discontinuous fiber-reinforced composites, with a unidirectional fiber orientation, can be estimated by the shear-lag model [18,19]:

(7) $$\sigma_{c}^{c}=\sigma_{c}^{f}V_{f}\left(1-\frac{l_{cr}}{2l_{f}}\right)+{\sigma^{\prime }}_{c}^{m}\left(1-V_{f}\right)\;\;\;\left(l\geq l_{cr}\right)$$

and

(8) $$\sigma_{c}^{c}=\frac{\tau_{y}}{d_{f}}V_{f}+\sigma_{c}^{m}\left(1-V_{f}\right)\;\;\;\left(l<l_{cr}\right)$$

where the superscripts c, f and m indicate the composite, fiber and matrix, respectively. $\sigma_{c}^{c}$ and $\sigma_{c}^{m}$ are UTS of the matrix and the matrix. ${\sigma^{\prime }}_{c}^{m}$ is the stress on the matrix when the reinforcing fibers fracture in the composite, $\tau_{y}$ is the shear yield stress of the matrix (almost equal to half the tensile yield stress of the matrix) and $l_{cr}$ is the critical length of the fiber, which determines which equation (Equations (7) or (8)) is used to estimate $\sigma_{c}^{c}$. $l_{cr}$ is the required minimum value for a perfect load transfer, such that the reinforcing fibers could ultimately be loaded up to their UTS values and fail during the loading of the composite. Therefore, the loading of the composite involves failure of the reinforcing fibers when $l\geq l_{cr}$. Hence, the critical length $l_{cr}$ is given as:

(9) $$l_{cr}=\frac{d\sigma_{c}^{f}}{2\tau_{y}}$$

Equations (7)–(9) are for discontinuous fiber-reinforced composites with unidirectionally oriented fibers. Therefore, the fiber orientation must be considered to accurately estimate the UTS of composites, which have a two-dimensional random fiber orientation.

Fukuda and Chou proposed the orientation factor, $C_{o}$, to account for the fiber orientations [20]. Equations (7) and (8) are thus modified to:

(10) $$\sigma_{c}^{c}=C_{0}\sigma_{c}^{f}V_{f}\left(1-\frac{l_{cr}}{2l_{f}}\right)+{\sigma^{\prime }}_{c}^{m}\left(1-V_{f}\right)\;\;\;\left(l\geq l_{cr}\right)$$

and

(11) $$\sigma_{c}^{c}=C_{0}V_{f}\frac{\tau_{y}}{d_{f}}+\sigma_{c}^{m}\left(1-V_{f}\right)\;\;\;\left(l<l_{cr}\right)$$

The probability density functions with respect to fiber orientation, g(θ), are assumed as:

(12) $$\{\begin{array}{cc} g\left(\theta \right)=\frac{\pi }{2\alpha }\cos\frac{\pi \theta }{2\alpha } & \left(0\leq \theta \leq \alpha \right)\\ g\left(\theta \right)=0 & \left(\alpha <\theta \right) \end{array}$$

where α is the maximum fiber orientation angle.

The orientation factor, $C_{o}$ is calculated as:

(13) $$\begin{array}{l} C_{0}=\frac{1}{16}[\frac{1}{1+q}\sin\left(\frac{\pi }{2}\left(1+q\right)\right)\\ \;+\frac{1}{1-q}\sin\left(\frac{\pi }{2}\left(1-q\right)\right)][\frac{3}{1+q}\sin\left(\frac{\pi }{2}\left(1+q\right)\right)\\ \;+\frac{3}{1-q}\sin\left(\frac{\pi }{2}\left(1-q\right)\right)+\frac{1}{1+3q}\sin\left(\frac{\pi }{2}\left(1+3q\right)\right)+\frac{1}{1-3q}\sin\left(\frac{\pi }{2}\left(1-3q\right)\right)] \end{array}$$

where

(14) $$q=\frac{2\alpha }{\pi }$$

## 4. Results and Discussion

### 4.1. Microstructure

**Figure 2:**
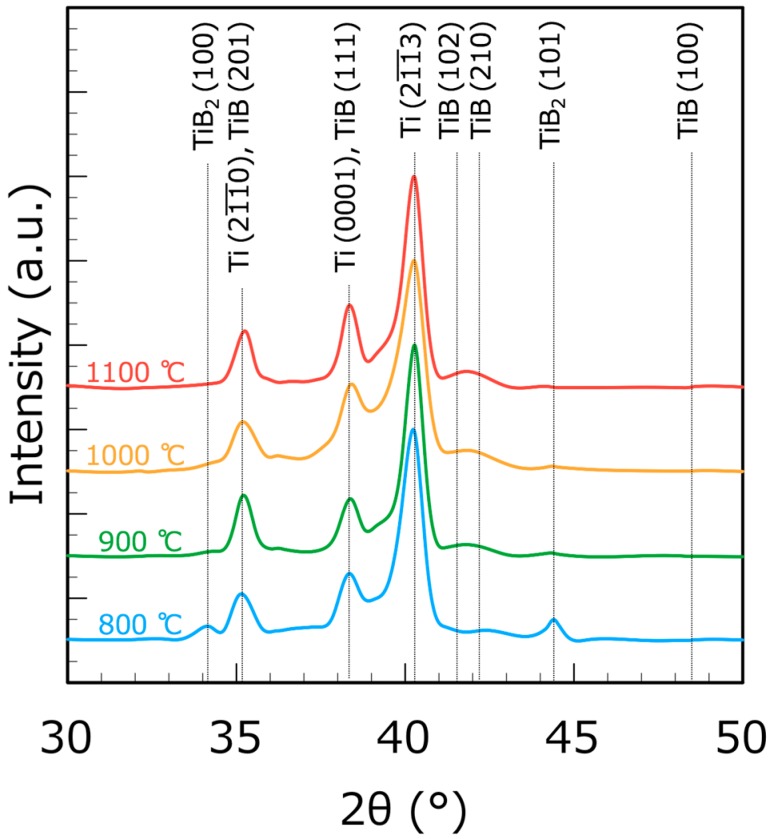
XRD patterns of SPS compacts (~12.5 vol.% TiB in the final composite) at each temperature.

Figure 2 shows the XRD patterns of the SPS compacts consolidated at each temperature. The peaks of the TiB_2_ (100) and (101) planes decreased, whereas the peaks of the TiB (102) and (210) planes increased between the SPS compacts at 800 and 900 °C, suggesting a dramatic progression of the reaction ${\mathrm{Ti}+\mathrm{TiB}}_{2}\to 2\mathrm{TiB}$ from 800 to 900 °C [21]. No peaks were observed for the TiB_2_ (100) and (101) planes in the SPS compacts at 1100 °C, even though detectable peaks were present in the SPS compacts at 900 and 1000 °C. Therefore, it seemed that TiB_2_ was completely transformed to TiB during SPS at 1100 °C for 30 min.

**Figure 3:**
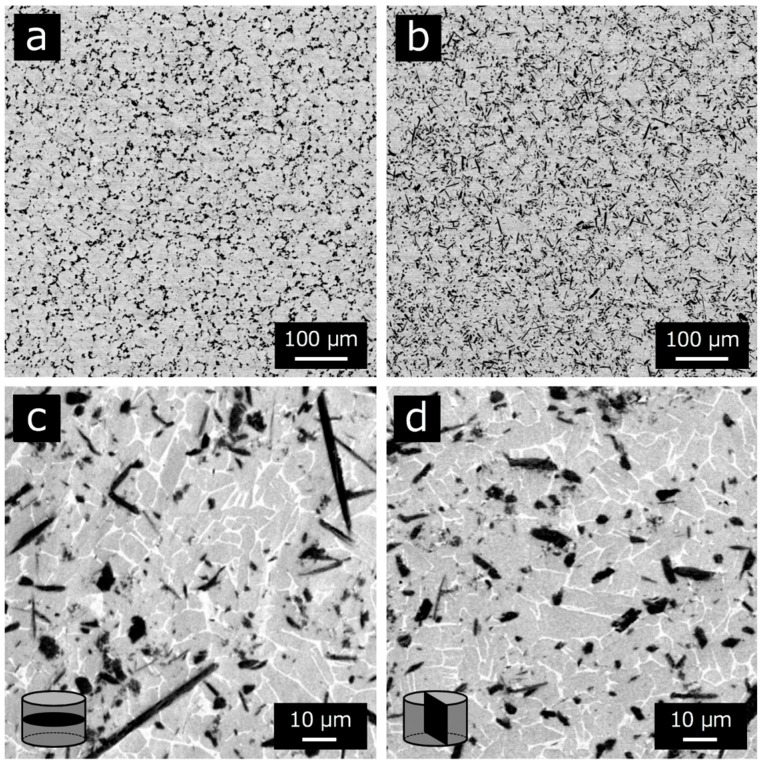
SEM images of SPS compacts consolidated for 30 min (~12.5 vol.% TiB in the final composite) at (**a**) 800 °C, (**b**) 1100 °C, (**c**) 1100 °C in direction perpendicular to the load (high magnification) and (**d**) 1100 °C in direction parallel to the load (high magnification).

**Figure 4:**
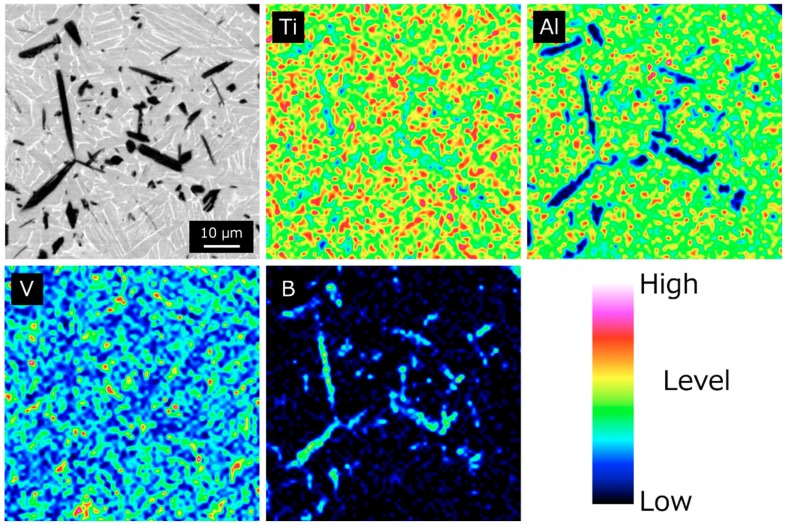
FE-EPMA results of SPS compact consolidated (~12.5 vol.% TiB in the final composite) at 1100 °C for 30 min.

Figure 3 shows the SEM images of the SPS compacts. No agglomeration of the particles and voids was observed in any of the SPS compacts. For the SPS compacts fabricated at 800 °C, the spherical TiB_2_ particles were dispersed in the particle boundaries of the Ti-6Al-4V alloy matrix (see Figure 3a). In contrast, needle-like crystals appeared in the Ti-6Al-4V alloy matrix in the SPS compacts fabricated at higher than 900 °C (see Figure 3b). These needle-like crystals were identified as TiB whiskers by the XRD patterns (see Figure 2) and FE-EPMA analysis (see Figure 4). The TiB whiskers tended to be oriented perpendicular to the load direction (i.e., two-dimensional random direction) in the matrix during the consolidation process under a uniaxially applied load (see Figure 3c,d). Moreover, the TiB whiskers were diffused inside the Ti-6Al-4V alloy matrix powder without an intermediate interface layer. 

We obtained the fully dense Ti6Al4V-TiB composites with no agglomeration of the TiB whiskers by SPS at 1100 °C for 30 min. The Ti-6Al-4V/TiB interface was intimate without an intermediate interfacial layer. The TiB whiskers tended to be oriented perpendicular to the loading direction in the Ti6Al4V-TiB composites, therefore it is highly expected that the TiB whiskers are oriented perpendicular to the loading direction in thin disc-shape Ti6Al4V-TiB compacts. Consequently, these Ti6Al4V-TiB composites thus allow us to discuss their tensile properties in comparison with the theoretical equations for discontinuous fiber-reinforced composites to reveal the strengthening mechanism of these Ti6Al4V-TiB composites.

### 4.2. Tensile Properties

**Figure 5:**
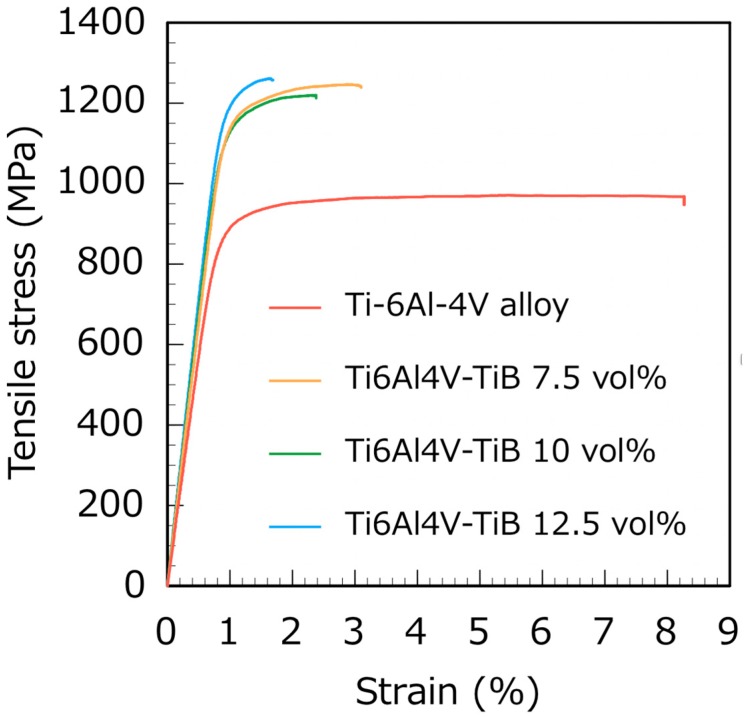
Typical stress-strain curves of Ti6Al4V-TiB composites.

**Figure 6:**
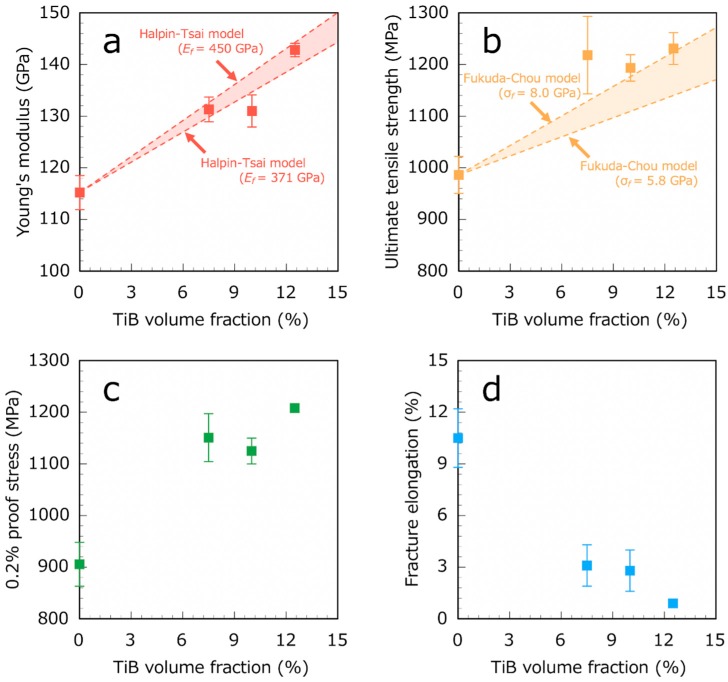
Tensile properties of Ti6Al4V-TiB composites versus TiB volume fraction for (**a**) Young’s modulus, (**b**) ultimate tensile strength, (**c**) 0.2% proof stress and (**d**) fracture elongation.

Figure 5 and Figure 6 show the typical stress-strain curves and the tensile properties versus the TiB volume fraction of the Ti6Al4V-TiB composites, respectively. The Young’s modulus, UTS and 0.2 proof stress of the Ti6Al4V-TiB composites all exhibited a proportional increase with the increase in TiB volume fraction. The Ti6Al4V-TiB 10 vol.% composite had a Young’s modulus of 130 GPa and an UTS of 1193 MPa. Furthermore, our Ti6Al4V-TiB composites demonstrated higher fracture elongation (2.8%) compared with those reported in previous studies due to the homogeneous dispersion of TiB whiskers in the Ti-6Al-4V alloy matrix [1]. 

To discuss the tensile properties of the Ti6Al4V-TiB composites with the theories for discontinuous fiber-reinforced composites, it is required that the homogeneous fiber dispersion in the matrix and perfect matrix/fiber interface contact without any intermediate layer are assumed in the theories. As mentioned above, it seems that the microstructure of our Ti6Al4V-TiB composites satisfies the applicable condition of these theories.

In this study, $E_{m}=115.2\;\mathrm{GPa}$ for the Ti-6Al-4V alloy. $E_{f}$ was assumed to be 371–450 GPa, taken from estimates of the Young’s modulus for TiB whiskers in previous studies [22,23,24]. The length and diameter of the fibers (TiB whiskers) were 40 and 2 μm, respectively, which were the average dimensions of the TiB whiskers observed in the SEM images. As shown in Figure 6a, the experimental Young’s modulus of the Ti6Al4V-TiB composites was consistent with the theoretical one estimated by the Halpin-Tsai model.

Furthermore, $\sigma_{cf}$, the UTS of the TiB whiskers is assumed to be 5.8–8.0 GPa, which has been estimated in a previous study [7]. $\sigma_{cm}$ is 986 MPa and $\tau_{y}$ is 452.5 MPa, which are estimated from the experimental UTS and half value of 0.2% proof stress (905 MPa) of the Ti-6Al-4V alloy, respectively. ${\sigma^{\prime }}_{c}^{m}$ is assumed to be $\sigma_{cm}$ = 986 MPa in this study because the fracture elongation of TiB whisker has not been reported yet. The length and diameter of the TiB whiskers are ~40 and ~2 μm, respectively, as mentioned above. Considering that our Ti6Al4V-TiB composites have the TiB orientation perpendicular to the loading direction in the Ti-6Al-4V alloy matrix, α=90°, thus yielding $C_{o}=0.463$ from Equation (13). As shown in Figure 6b, the experimental UTS of the Ti6Al4V-TiB composites was consistent with the theoretical one estimated by the Fukuda-Chou model.

**Figure 7:**
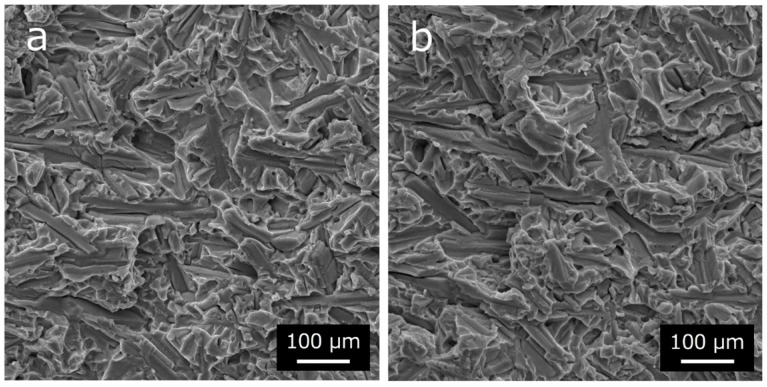
Pair of SEM fractographs for Ti6Al4V−12.5 vol.% TiB composites; (**a**) normal image and (**b**) mirror-reversed image of opposite fracture surface of (**a**).

The elongation of our Ti6Al4V-TiB composites decreased in the presence of TiB whiskers (see Figure 6d). Figure 7 shows the pair of SEM fractographs for the Ti6Al4V-TiB 12.5 vol.% composites. Note that Figure 7b is the mirror-reversed image of the opposite fracture surface of Figure 7a. The fractured TiB whiskers were exposed on both sides of the flat fracture surface. Moreover, the percentage of exposed fractured TiB whiskers was larger than the TiB volume fraction. This result indicates that the crack selectively occurred from inside TiB whicker under tensile load, or selectively propagated through the TiB whiskers during the failure of the Ti6Al4V-TiB composites [25,26]. It is implied that this selective crack occasion or propagation inside the TiB whiskers occurs due to the drastic decrease in the elongation of the Ti6Al4V-TiB composites. Therefore, it seems that the smaller TiB whiskers probably inhibit the immediate failure of the Ti6Al4V-TiB composites.

## 5. Conclusions

We fabricated fully dense Ti6Al4V-TiB composites by SPS. These Ti6Al4V-TiB composites had a homogeneous dispersion of TiB whiskers in a Ti-6Al-4V alloy matrix. The Ti-6Al-4V/TiB interface was intimate without an intermediate interfacial layer. Furthermore, the TiB whiskers tended to be oriented perpendicular to the loading direction in Ti6Al4V-TiB composites. This microstructure was suitable to discuss the tensile properties of the Ti6Al4V-TiB composites with the theories for discontinuous fiber-reinforced composites.

The Young’s modulus, UTS and 0.2% proof stress of the Ti6Al4V-TiB composites all increased with the increase in TiB volume fraction. The Ti6Al4V-TiB 10 vol.% composite yielded a Young’s modulus of 130 GPa, a UTS of 1193 MPa and a fracture elongation of 2.8%. The experimental Young’s modulus and UTS of the Ti6Al4V-TiB composites were consistent with the theoretical values estimated by the Halpin-Tsai and Fukuda-Chou models, which are the theoretical equations for discontinuous fiber-reinforced composites. The good agreement of our experimental results with these theories indicates that the TiB whiskers behave as discontinuous fibers in the Ti-6Al-4V alloy matrix. 

The elongation of the Ti6Al4V-TiB composites was drastically decreased by selective crack propagation inside the TiB whiskers during the failure of Ti6Al4V-TiB composites. It seems that the miniaturization of the TiB whisker size allowed both the high tensile strength and plastic deformability of the Ti6Al4V-TiB composites obtained.
